# Supplementary material for: Discovery of Eicosapentaenoic Acid Esters of Hydroxy Fatty Acids as Potent Nrf2 Activators
Source: Antioxidants (Basel). 2020 May 8;9(5):397. doi: 10.3390/antiox9050397 (PMC7278747; doi:10.3390/antiox9050397)

Supporting material for

Discovery of Eicosapentaenoic Acid Esters of Hydroxy Fatty Acids as Potent Nrf2 activators

Siddabasave Gowda B. Gowda [1], Hirotoshi Fuda [1], Takayuki Tsukui [2], Hitoshi Chiba [2], and Shu-Ping Hui*[1]

1. Faculty of Health Sciences, Hokkaido University, Kita-12, Nishi-5, Kita-Ku, Sapporo 060-0812, Japan
2. Department of Nutrition, Sapporo University of Health Sciences, Nakanuma Nishi-4-3-1-15, Higashi-Ku, Sapporo 007-0894, Japan.

Contents:

1. Figure S1: **S1**
2. Figure S2: **S2**
3. Figure S3: **S3**
4. ^{1}H and ^{13}C-NMR spectra of FAHFAs: **S4-S9**

Figure S1: Reporter gene (luciferase) assay results of oleic acid, linoleic acid esters of 12-HSA and 12-HOA and free fatty acids (EPA and 12-HSA)

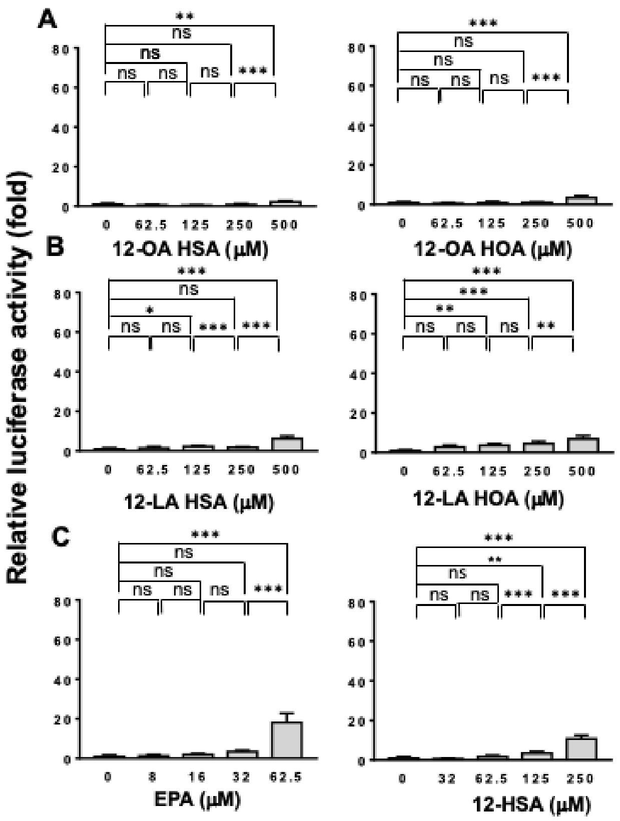

Figure S2: Relative expression of DDIT3 (CHOP) (NM001195053) in response to 12-EPAHSA treatment. **p<0.01, (one-way ANOVA) (n=6).

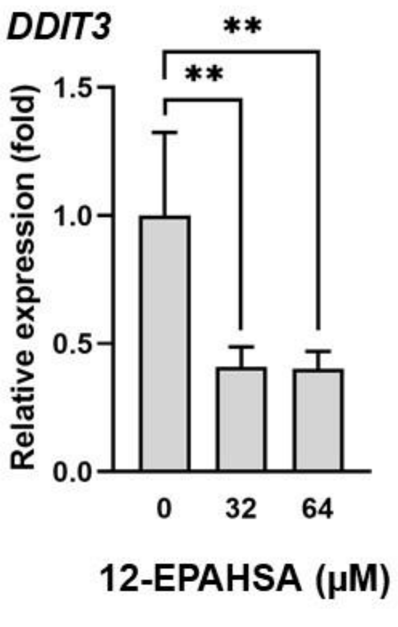

Figure S3: Relative intensity folds of oxidative stress induced by H_2O_2 and effect of 12-EPAHSA treatment. ***p<0.001, (one-way ANOVA) (n=6 for control and n=4 for H_2O_2). The cellular oxidative stress due to H_2O_2-induced ROS was measured by the dichlorofluorescein-diacetate (DCFH-DA) assay with the protocol established in our laboratory [17-18]. A condition of cellular stress in HepG2 cells is evoked by treating 0.25 mM H_2O_2 for 30 min, and fluorescence intensity was read at 485 (excitation) and 525 (emission) nm using Wallac 1420 ARVO Mx plate reader (PerkinElmer, Japan).

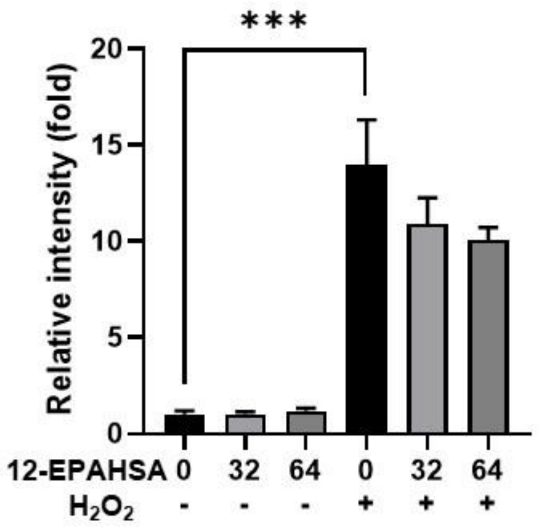

3. ^{1}H and ^{13}C-NMR spectra of FAHFAs

^{1}H and ^{13}C-NMR of 12-OAHSA (2)

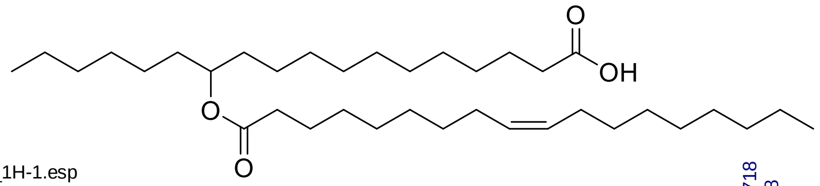

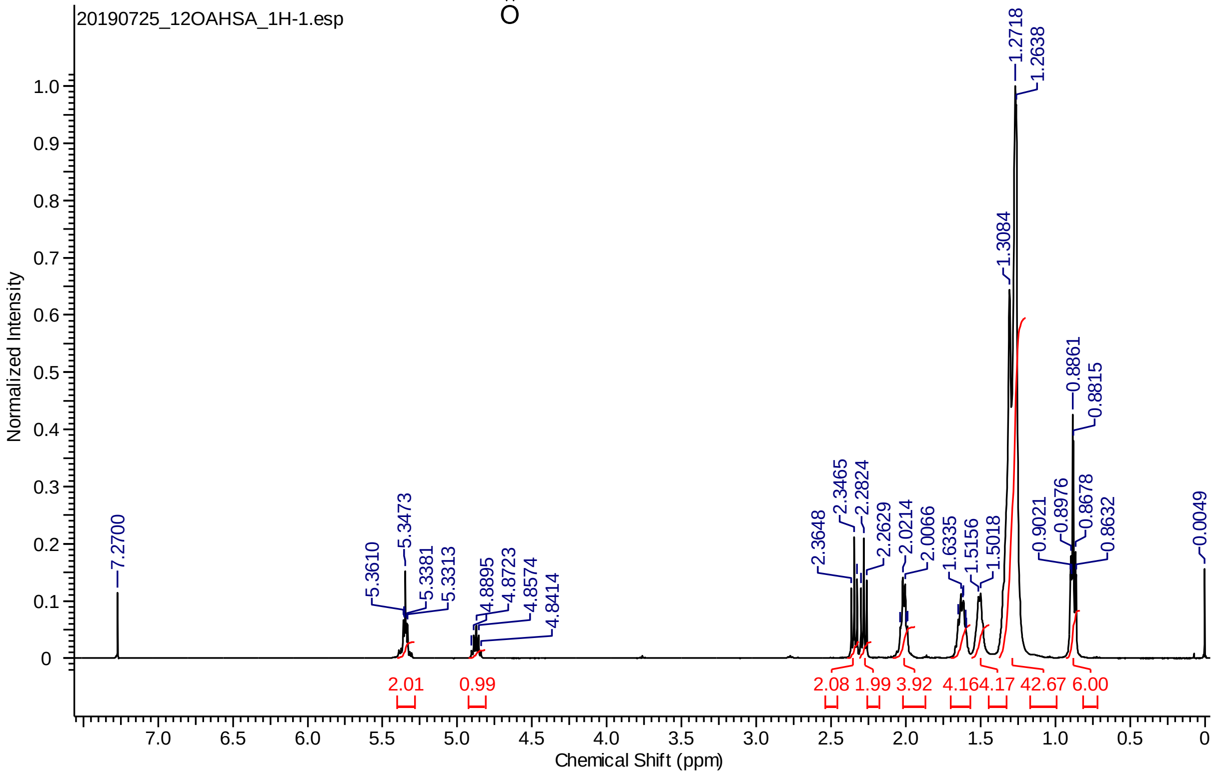

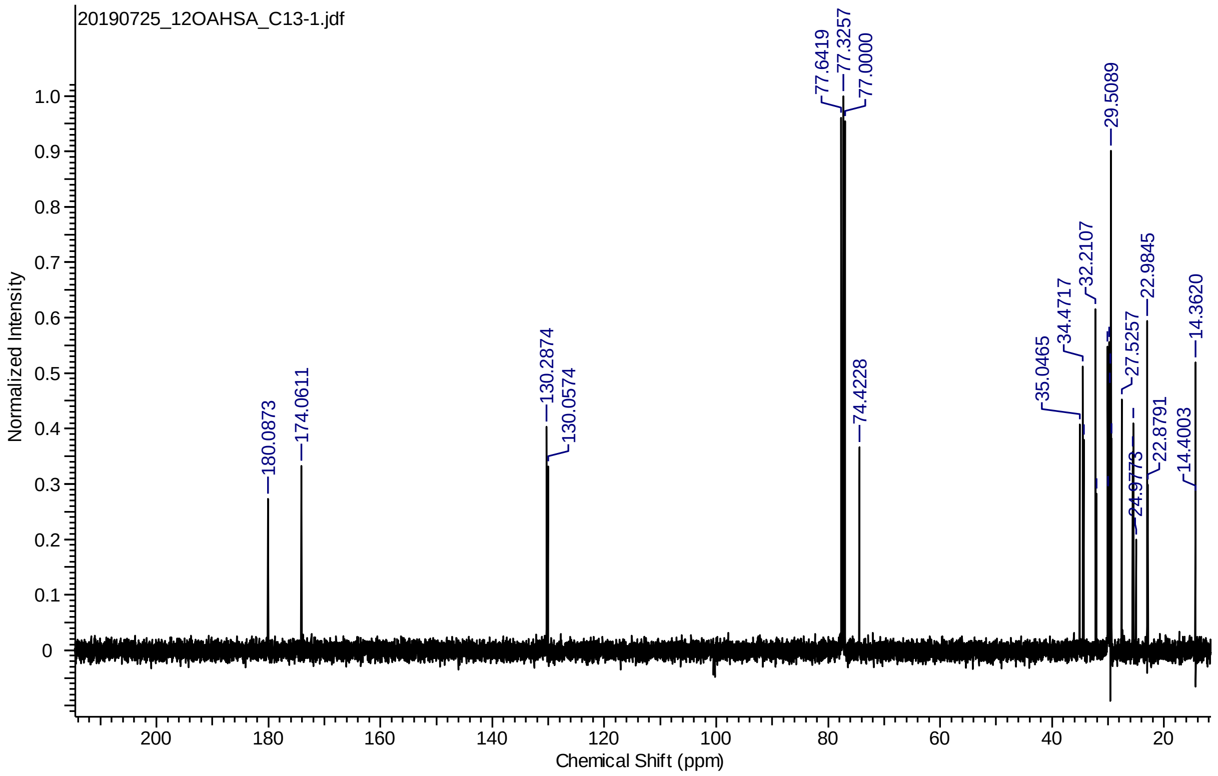

^{1}H and ^{13}C-NMR of 12-OAHOA (2a)

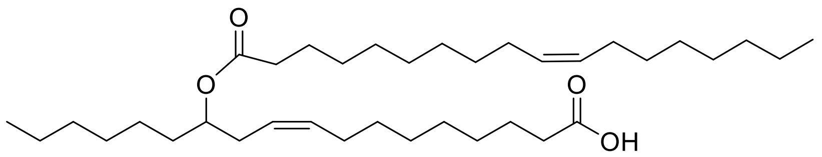

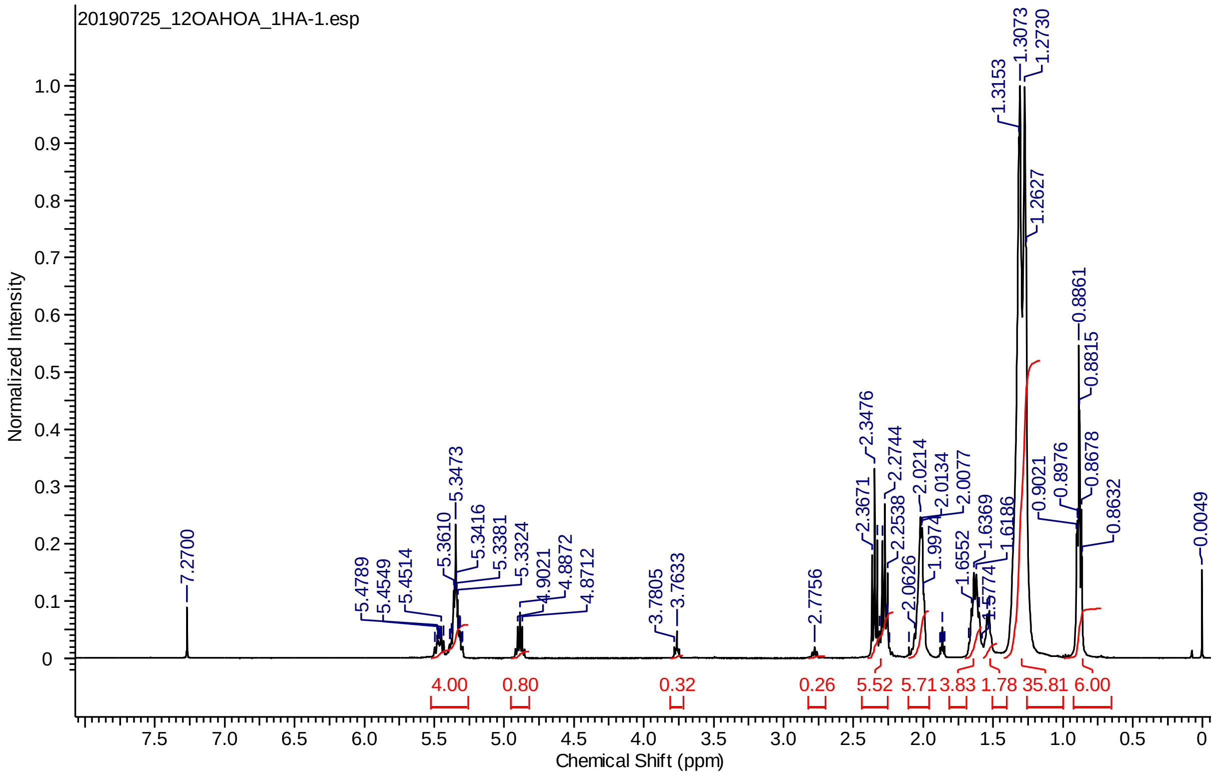

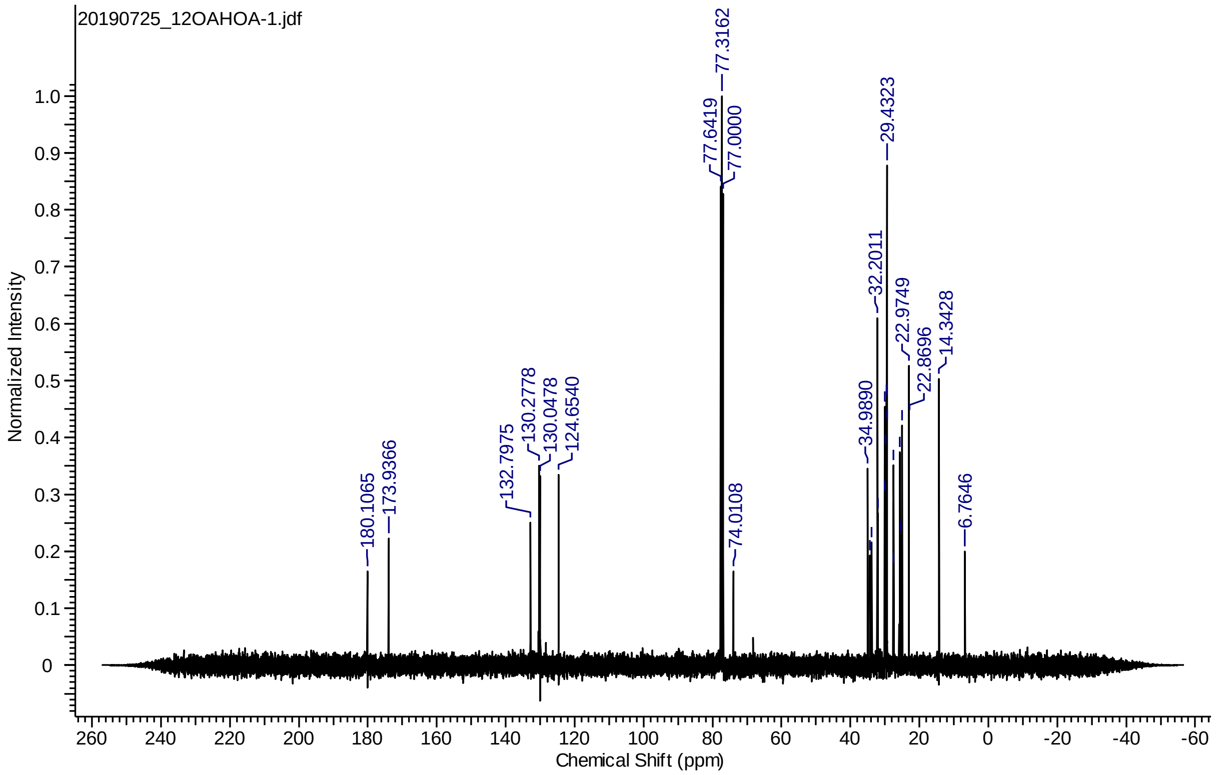

^{1}H and ^{13}C-NMR of 12-LAHSA (3)

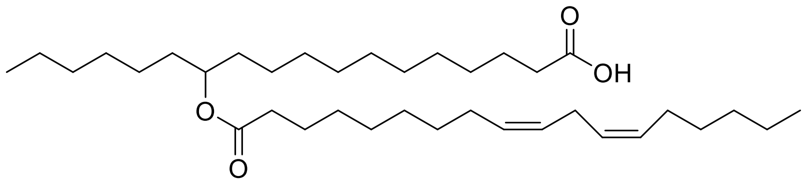

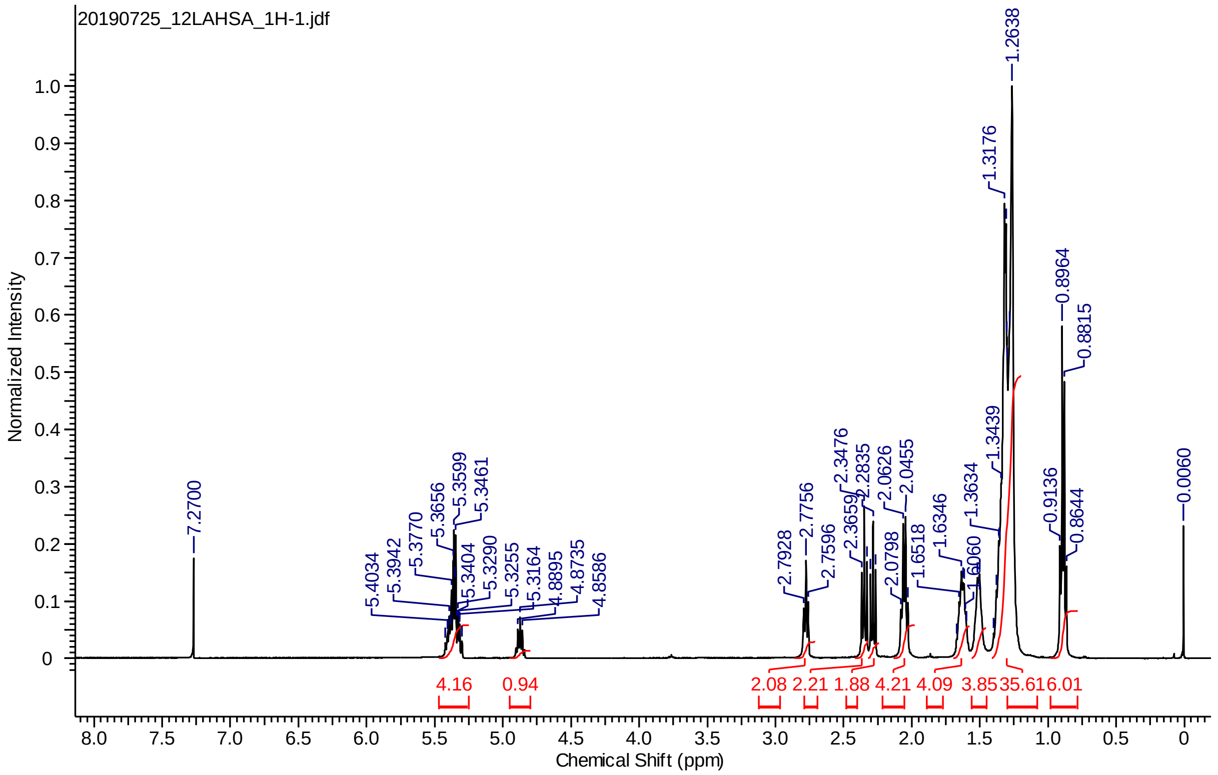

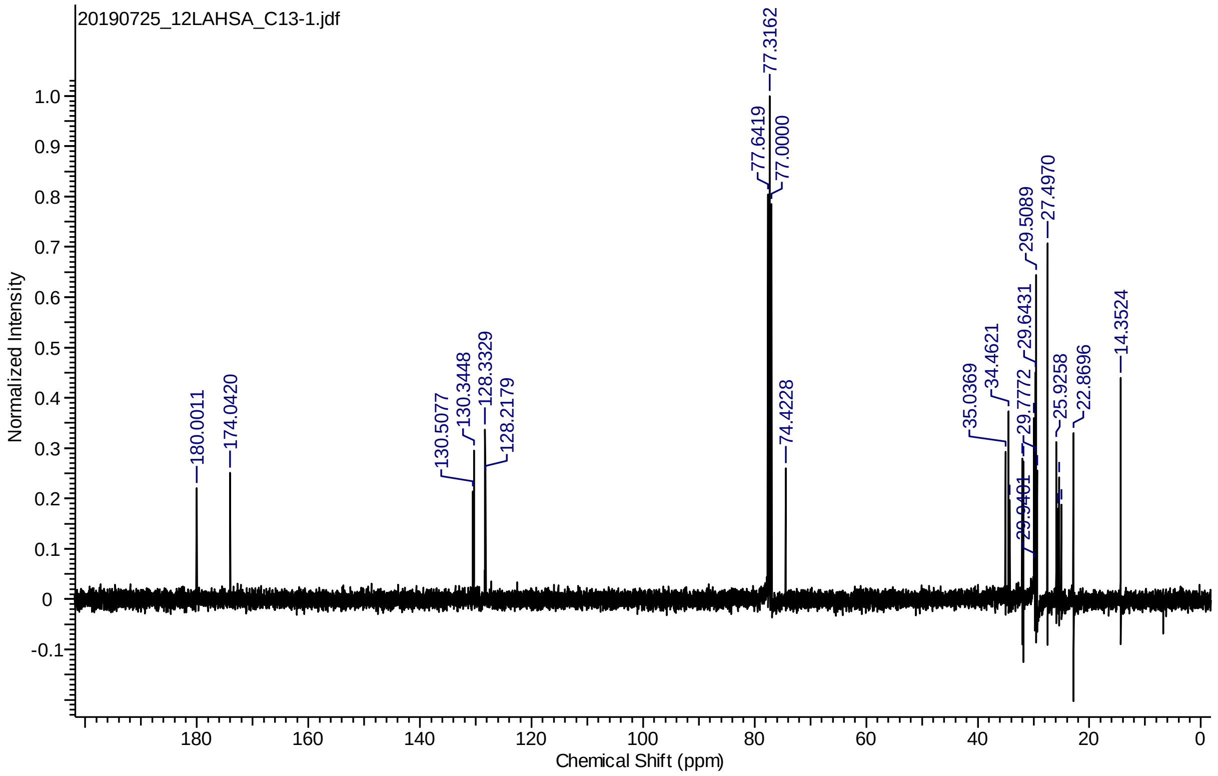

^{1}H and ^{13}C-NMR of 12-LAHOA (3a)

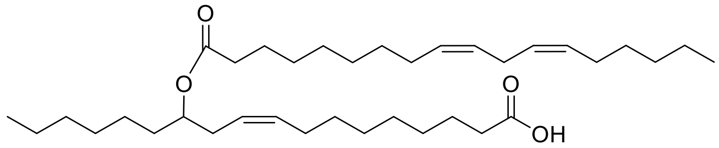

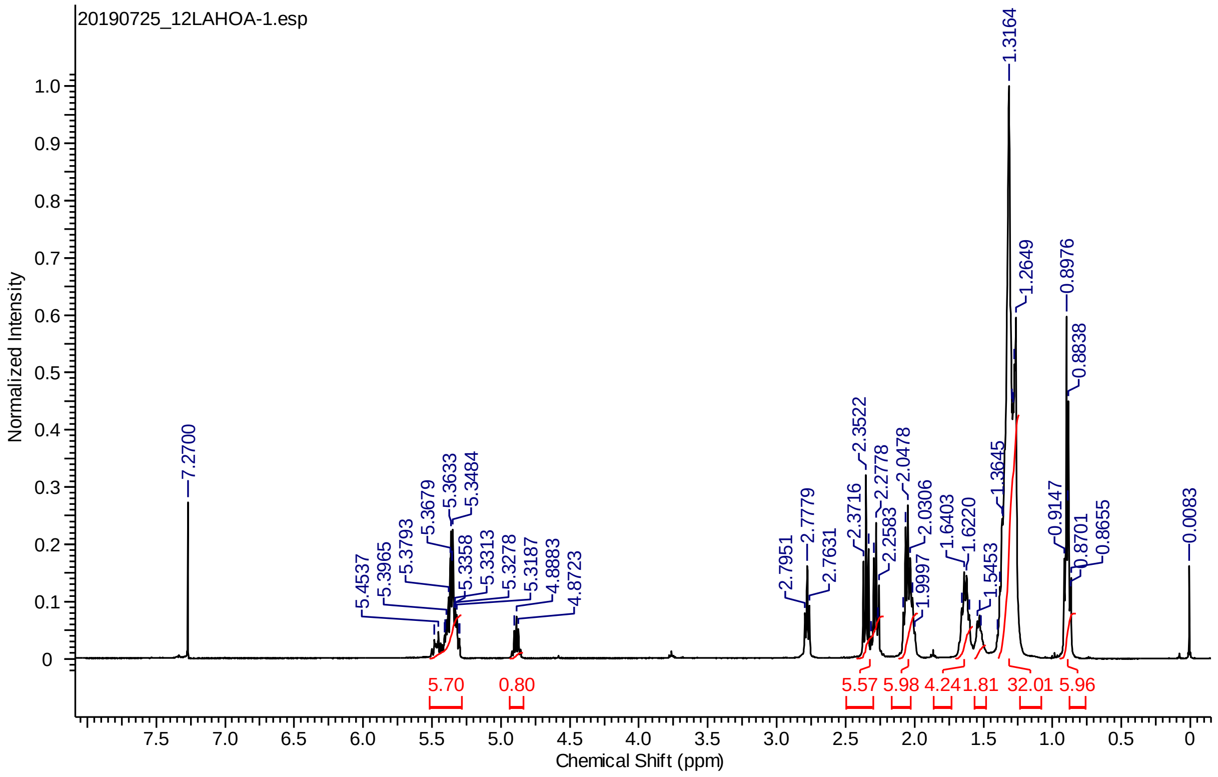

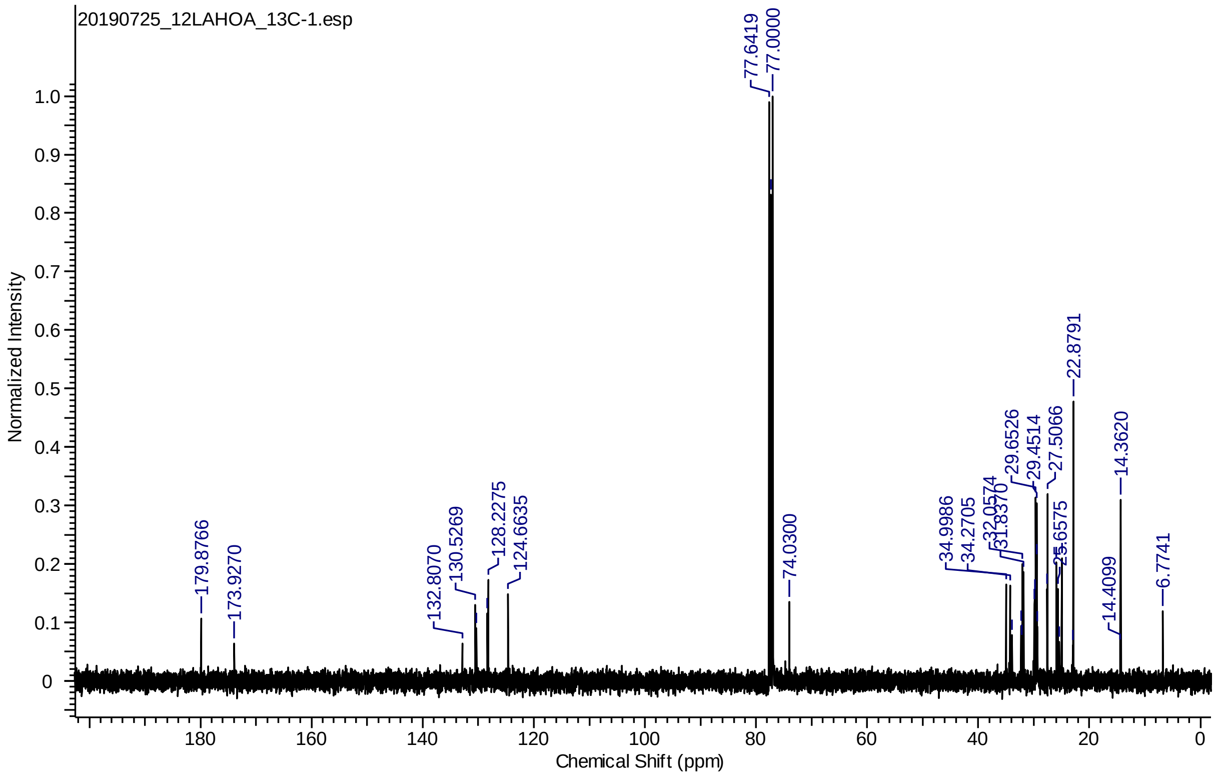

^{1}H and ^{13}C-NMR of 12-EPAHSA (4)

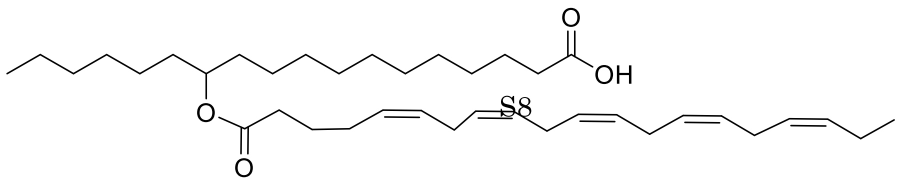

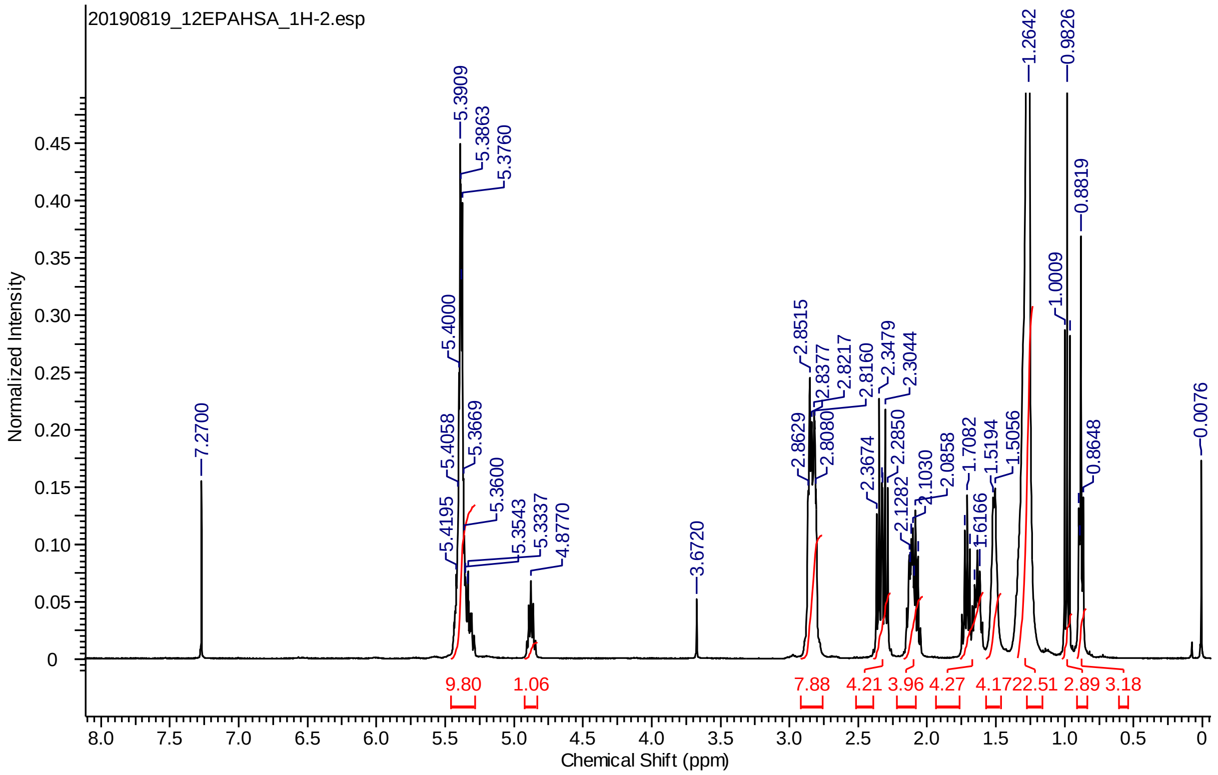
20190819_12EPAHSA_1H-2.esp
Normalized Intensity
Chemical Shift (ppm)
7.2700
5.4195
5.4058
5.4000
5.3909
5.3863
5.3760
5.3669
5.3600
5.3543
5.3337
4.8770
3.6720
2.8629
2.8515
2.8377
2.8217
2.8160
2.8080
2.3674
2.3479
2.3044
2.2850
2.1282
2.1030
2.0858
1.7082
1.6166
1.5194
1.5056
1.2642
1.0009
0.9826
0.8819
0.8648
-0.0076
9.80
1.06
7.88
4.21
3.96
4.27
4.17
22.51
2.89
3.18

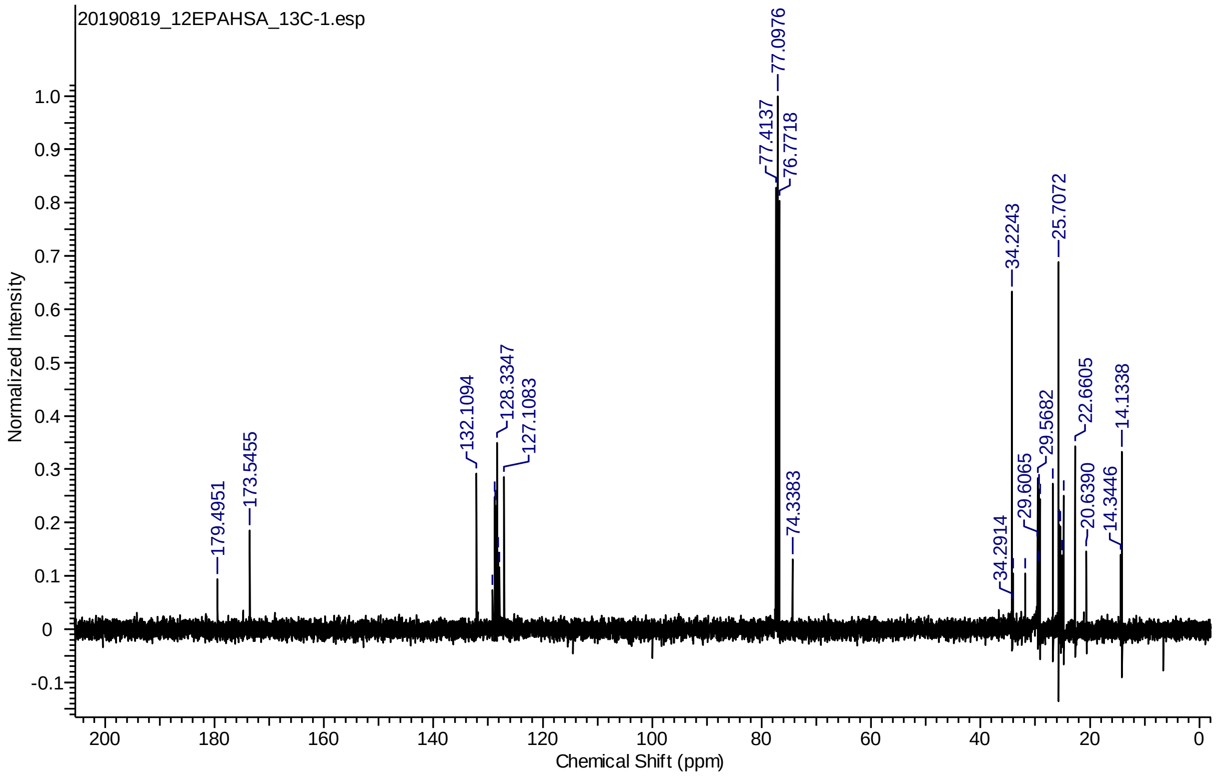
20190819_12EPAHSA_13C-1.esp
Normalized Intensity
Chemical Shift (ppm)
179.4951
173.5455
132.1094
128.3347
127.1083
77.4137
77.0976
76.7718
74.3383
34.2914
34.2243
29.6065
29.5682
25.7072
22.6605
20.6390
14.3446
14.1338

^{1}H and ^{13}C-NMR of 12-EPAHOA (4a)

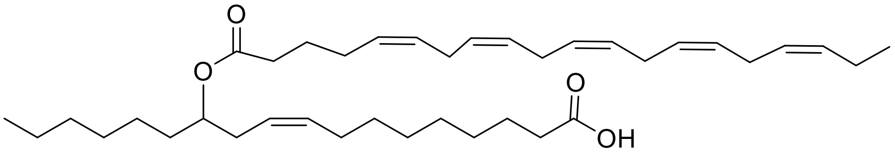

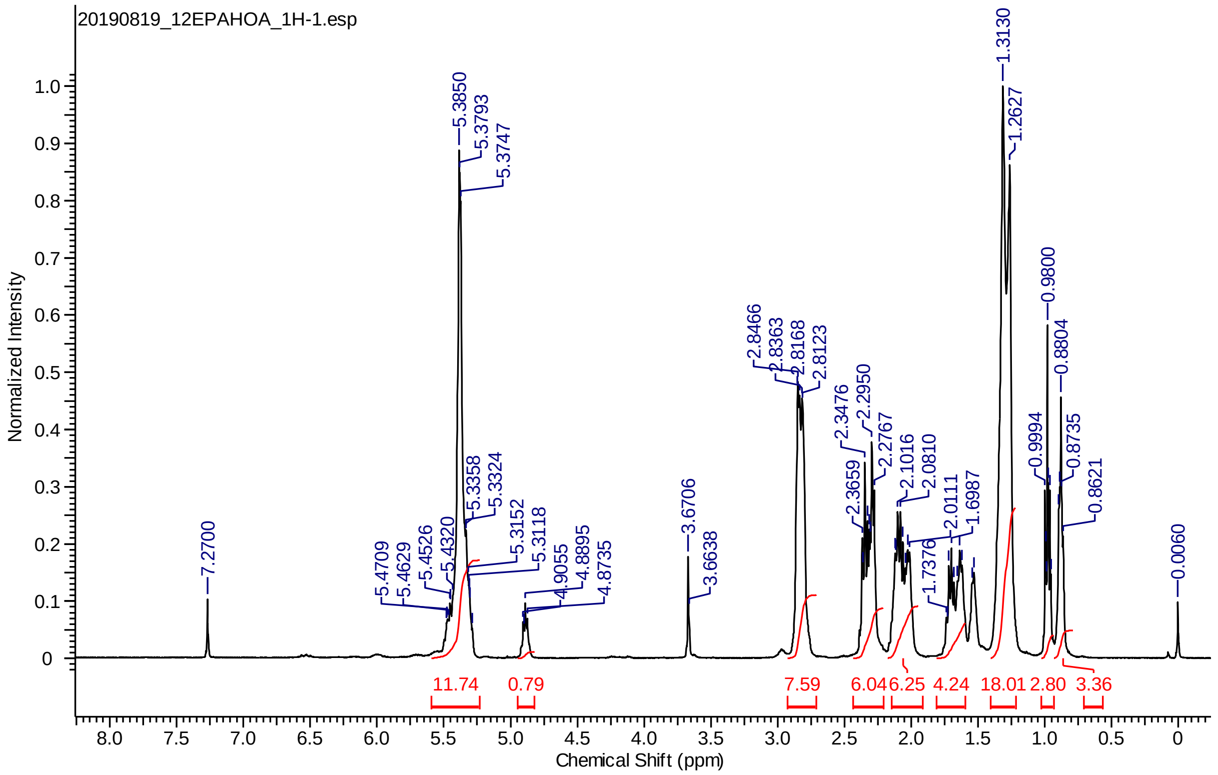

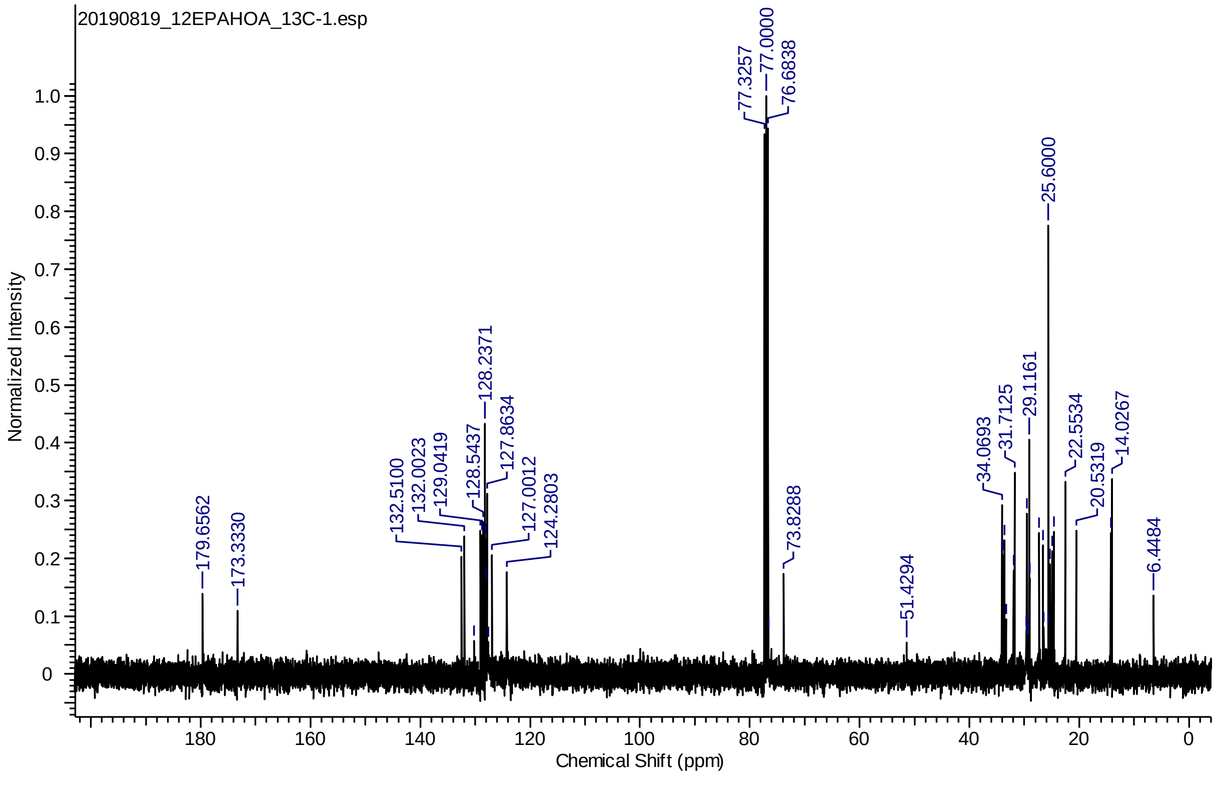

Supplement: Supplementary file 1 [file antioxidants-09-00397-s001.pdf]
